# CO-Reductive and O_2_-Oxidative Annealing Assisted Surface Restructure and Corresponding Formic Acid Oxidation Performance of PdPt and PdRuPt Nanocatalysts

**DOI:** 10.1038/s41598-020-65393-3

**Published:** 2020-05-21

**Authors:** Dinesh Bhalothia, Tzu-Hsi Huang, Pai-Hung Chou, Po-Chun Chen, Kuan-Wen Wang, Tsan-Yao Chen

**Affiliations:** 1 Department of Engineering and System Science, National Tsing Hua University, Hsinchu, 30013 Taiwan; 2 Institute of Materials Science and Engineering, National Central University, Taoyuan City, 32001 Taiwan; 3 Department of Materials and Mineral Resources Engineering, National Taipei University of Technology, Taipei, 10608 Taiwan; 4 Hierarchical Green-Energy Materials (Hi-GEM) Research Centre, National Cheng Kung University, Tainan, 70101 Taiwan

**Keywords:** Energy science and technology, Fuel cells, Energy, Applied physics, Chemical physics

## Abstract

Formic acid oxidation reaction (FAOR) at anode counterpart incurs at substantial high overpotential, limiting the power output efficiency of direct formic acid fuel cells (DFAFCs). Despite intense research, the lack of high-performance nanocatalysts (NCs) for FAOR remains a challenge in realizing DFAFC technologies. To surmount the overpotential losses, it is desirable to have NCs to trigger the FAOR as close to the reversible conditions (i.e. with over-potential loss as close to zero as possible). Herein, Pd-based binary and ternary NCs consisting of PdPt and PdRuPt have been synthesized via the polyol reduction method on the carbon support. As prepared PdPt and PdRuPt NCs were further subjected to heat treatment (annealed) in CO (namely PdPt-CO and PdRuPt-CO) and O_2_ (namely PdPt-O_2_ and PdRuPt-O_2_) atmosphere at 473 K temperature. By cross-referencing results of electron microscopy and X-ray spectroscopy together with electrochemical analysis, the effects of heat treatment under CO-reductive and O_2_-oxidative conditions towards FAOR were schematically elucidated. Of special relevance, the mass activity (MA) of PdPt-CO, PdPt-O_2_, PdRuPt-CO, and PdRuPt-O_2_ NCs is 1.7/2.0, 1.3/2.2, 1.1/5.5, and 0.9/4.7 Amg^−1^ in the anodic/cathodic scan, respectively, which is 2~4-folds improved comparative to of as-prepared PdPt (1.0/1.9 Amg^−1^ in anodic/cathodic scan, respectively) and PdRuPt (0.9/1.4 Amg^−1^ in anodic/cathodic scan, respectively) NCs. Meanwhile, after chronoamperometric (CA) stability test up to 2000 s, PdPt-CO (72 mAmg^−1^) and PdRuPt-CO (213 mAmg^−1^) NCs exhibit higher MA compared to as-prepared PdPt (54 mAmg^−1^) and PdRuPt (62 mAmg^−1^) NCs, which is attributed to the increase of surface Pt composition, especially for PdRuPt-CO NC. Besides, the stability of PdPt-O_2_ (15 mAmg^−1^) and PdRuPt-O_2_ (22 mAmg^−1^) NCs is deteriorated as compared to that of as-prepared NCs due to severe oxidation in O_2_ atmosphere. Of utmost importance, we developed a ternary PdRuPt catalyst with ultra-low Pt content (~2 wt.%) and significantly improved FAOR performance than pure Pt catalysts. Moreover, we demonstrated that the FAOR performance can be further enhanced by more than 30% via a unique CO annealing treatment.

## Introduction

Severe global energy crisis coupled with adverse climatic issues have ignited great interest in the implementation of the sustainable energy economy. In this quest, fuel cells have emerged as potential energy conversion devices without increasing the carbon footprints in nature. Among these, direct formic acid fuel cells (DFAFCs) have gained more attention owing to their promising properties such as high open-circuit potential (1.45 V) and energy conversion efficiency (theoretical conversion efficiency of DFAFCs is reached to 106%), limited fuel crossover effects and lower toxicity^1–4^. Despite their great merits, the commercial exploitation of DFAFCs is strongly hampered by intrinsically sluggish kinetics of formic acid oxidation reaction (FAOR) at the anode^5^. Although, Pt-based nanocatalysts (NCs) are frequently employed to trigger FAOR kinetics, however, excessively strong adsorption of intermediate products (i.e. CO poisoning) limits the widespread application of Pt-based NCs because it causes not only deceleration the FAOR kinetics but also high anode overpotential^6–8^. Besides, high material cost and limited reserves of Pt in the crust are severe issues detaining the market potential of DFAFCs^9^. Consequently, development of NCs capable of providing high current densities at lower overpotential together with lower Pt-dosage and maximizing Pt utilization is urgent. Recently, multi-directional efforts have been devoted to improving Pt-utilization. To this end, transition metal additives in form of Pt-M alloys^10,11^ (where M represents non-Pt metals), M-core@Pt-shell nanostructures^12,13^, nanowires^14,15^, nano-rods^16,17^, nano-plates^18^, nano-dendrites^19,20^, nano-frames^21,22^, nano-chains^23^, nano-cages^24^, nanocubes^25^ etc. have been demonstrated to achieve desired geometry in NC design with appropriate balance between catalytic performance and noble-metal dosage. Moreover, particle size^26^, the morphology^27^ composition of NC^28^ and nature of support materials^29^ also influence the FAOR kinetics and recent studies have demonstrated significantly enhanced catalytic performance via changing these entities. Although the aforementioned strategies provide enormous approaches to design omnipotent FAOR catalyst, however, the last objective has remained elusive

It is undeniable fact that Pt is by far the first choice to boost FAOR kinetics. In this aspect, to further explore the next generation NCs with low Pt-content and high catalytic performance (structural reliability and FAOR activity), alloying Pt with Pd is a possible combination^30,31^. Pd holds a majority of physicochemical properties similar to those of Pt and relatively cheap. Moreover, Pd is less susceptible to poisoning through surface-adsorbed intermediates (such as CO) at low potentials^32,33^. Therefore PdPt bimetallic NCs have been intensively investigated and exhibited high catalytic activity towards FAOR as compared to that of pure Pt or Pd nanoparticles^34^. Although Pd has been confirmed to be a potential alternative to catalyze FAOR and can greatly enhance the FAOR performance when decorated with Pt-atoms, however, CO tolerance and high cost of Pd are still major obstacles for their commercial viability. To overcome these existing issues, decorating Ru at interface and surface of Pd or Pt NCs offers electronic, geometric and compositional effects that can be used to tune catalytic active sites, leads not only improved activity and cost reduction but better CO tolerance than commercial catalysts toward FAOR^35^.. In Ru-based bimetallic NCs (e.g. RuPt), the bifunctional mechanism plays an important role to facilitate the intermediate reactions (i.e. CO_ads_ to CO_2_), as shown in the following equations:^36^

1 $${\rm{Ru}}^\circ +{{\rm{H}}}_{2}{\rm{O}}\to {\rm{Ru}}-{\rm{OH}}+{{\rm{H}}}^{+}+{{\rm{e}}}^{-}$$

2 $${\rm{Pt}}-{\rm{CO}}+{\rm{Ru}}-{\rm{OH}}\to {\rm{Pt}}^\circ +{\rm{Ru}}^\circ +{{\rm{CO}}}_{2}+{{\rm{H}}}^{+}+{{\rm{e}}}^{-}$$

Aforementioned arguments inspired us to further tune the geometric and electronic properties of Pd-based NCs for enhanced FAOR performance (both activity and CO-tolerance). Herein, we synthesized Pd-based bimetallic (PdPt) and tri-metallic (PdRuPt) NCs via polyol reduction method. As prepared NCs were further subjected to annealing at 473 K temperature in CO (namely PdPt-CO and PdRuPt-CO) and O_2_ (namely PdPt-O_2_ and PdRuPt-O_2_) atmosphere. Compared with the mass activity (MA) of as-prepared PdPt (1.0/1.9 Amg^−1^ in anodic/cathodic scan, respectively) and PdRuPt (0.9/1.4 Amg^−1^ in anodic/cathodic scan, respectively) NCs, the MA of PdPt-CO, PdPt-O_2_, PdRuPt-CO, and PdRuPt-O_2_ NCs is 1.7/2.0, 1.3/2.2, 1.1/5.5, and 0.9/4.7 Amg^−1^ in the anodic/cathodic scan, respectively. By cross-referencing results of X-ray photoelectron spectroscopic (XPS) and CO stripping, we demonstrated that due to small change in surface composition, the MA of post-annealed PdPt NCs (i.e. PdPt-CO and PdPt-O_2_) did not change significantly as compared to pristine PdPt NC. Whereas, the drastic enhancement in FAOR performance of post annealed PdRuPt NCs during both CO and O_2_ treatments is attributed to the significant change in surface compositions. Moreover, after chronoamperometric stability test over the 2000s, CO treated PdPt (72 mAmg^−1^) and PdRuPt (213 mAmg^−1^) NCs show higher MA as compared to that of O_2_ treated PdPt (15 mAmg^−1^) and PdRuPt (22 mAmg^−1^) NCs. Such behavior is obvious due to severe oxidation.

## Results and Discussion

### Surface morphology and crystal structure

**Figure 1:**
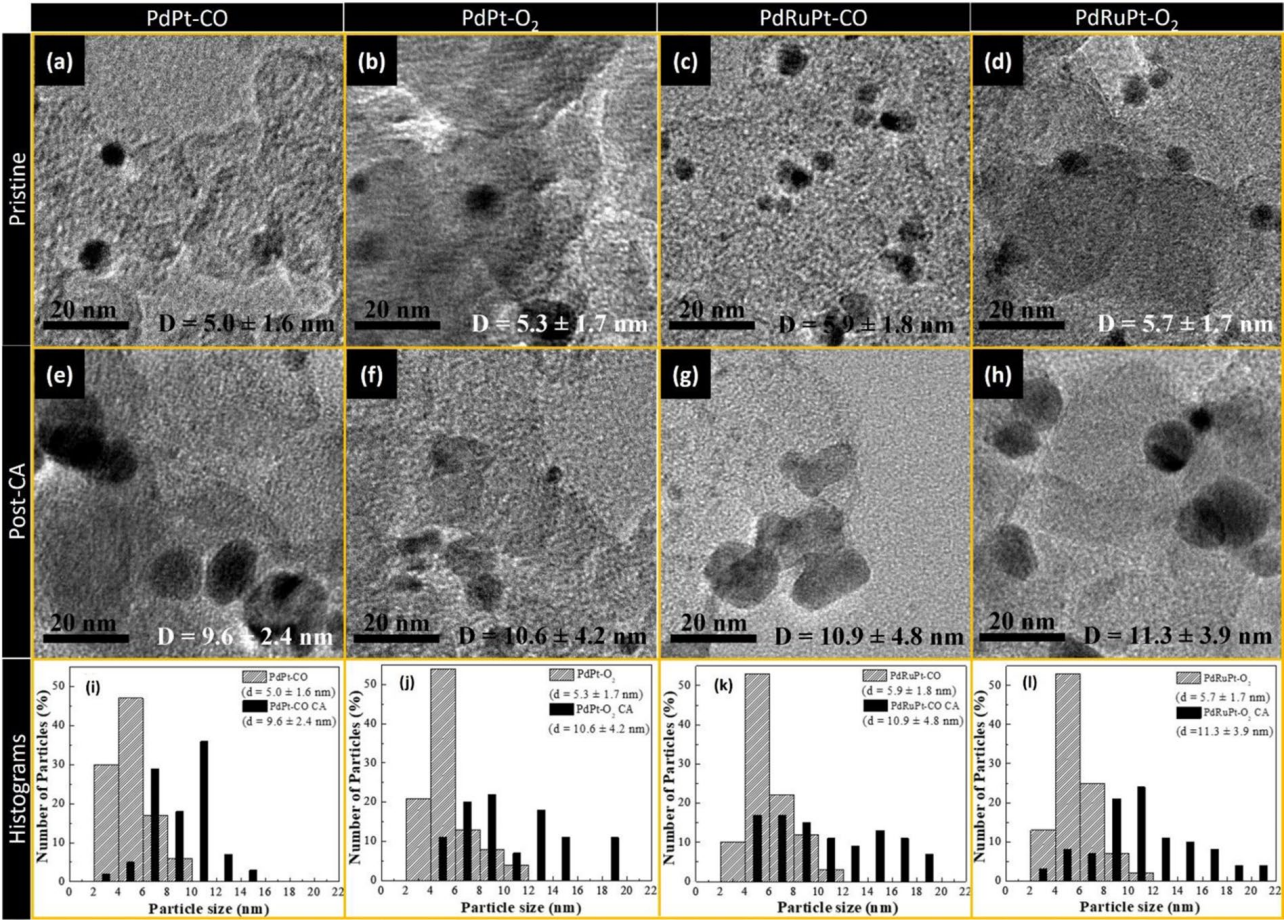
HRTEM images of before (**a**) PdPt-CO, (**b**) PdPt-O_2_, (**c**) PdRuPt-CO and (**d**) PdRuPt-O_2_ NCs, and after chronoamperometric stability test (**e**) PdPt-CO, (**f**) PdPt-O_2_, (**g**) PdRuPt-CO and (**h**) PdRuPt-O_2_ NCs. The particle-size distribution histograms of pristine and post chronoamperometric stability test are depicted in (**i**) PdPt-CO, (**j**) PdPt-O2, (**k**) PdRuPt-CO and (**l**) PdRuPt-O2 NCs.

The morphologies and the particle-size distribution histograms for PdPt-CO, PdPt-O_2_, PdRuPt-CO and PdRuPt-O_2_ before and after CA stability tests were determined by HRTEM characterization and shown in Fig. 1. Accordingly, the average particle size of PdPt-CO, PdPt-O_2_, PdRuPt-CO and PdRuPt-O_2_ is about 5.0 ± 1.7, 5.3 ± 1.8, 5.9 ± 1.8, and 5.7 ± 1.7 nm, respectively. To further clarify the effects of heat treatment on surface morphology and particle size, HRTEM images of as-prepared PdPt and PdRuPt NCs have been shown in Figure S1. Compared to as-prepared NCs (Figure S1**)**, aggregation of NCs is observed during the heating process. Besides, all heat-treated NCs show further aggregation after the CA durability test.

**Figure 2:**
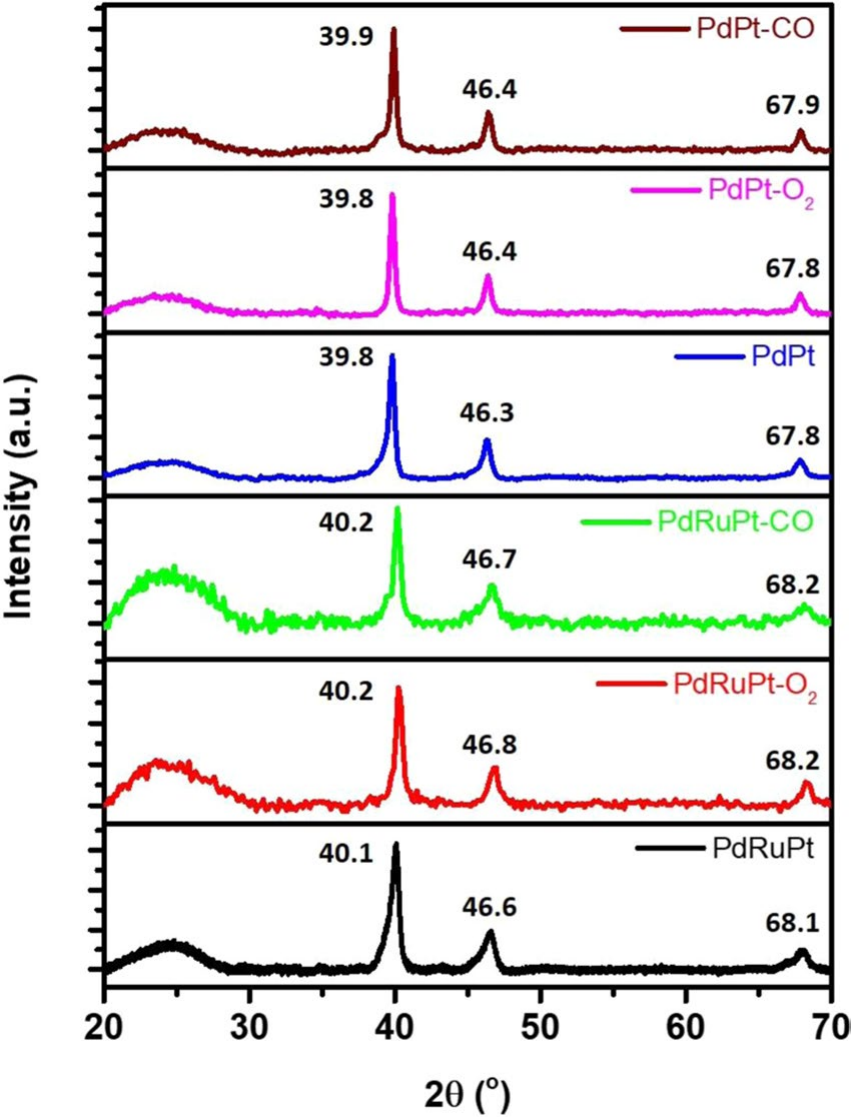
XRD patterns of as-prepared and post-annealed (CO and O_2_ atmosphere) PdPt and PdRuPt NCs.

The XRD patterns of post-annealed experimental NCs (i.e. PdPt-CO/O_2_ and PdRuPt-CO/O_2_) compared with as-prepared NCs (i.e. PdPt and PdRuPt) are displayed in Fig. 2. Accordingly, the diffraction peak centered nearly at ~25° is corresponding to XC-72R carbon support. The diffraction signals of binary PdPt, PdPt-CO and PdPt-O_2_ NCs are located between those of metallic face-centred cubic (FCC) Pd (JCPDS 87–0643) and Pt (JCPDS 87–0646). Whereas the diffraction peaks of different PdRuPt, PdRuPt-CO and PdRuPt-O_2_ NCs are located between those of Pt, Pd and Ru (JCPDS 88–2333), suggesting the formation of PtPd and PtPdRu homogeneous structures, respectively. The heat treatment in the O_2_ and CO atmosphere did not change the bulk structure and the peak positions are almost the same. Moreover, the lattice parameters calculated from (220) planes for PdPt, PdPt-CO, PdPt-O_2_, PdRuPt, PdRuPt-CO, and PdRuPtO_2_) are 3.903, 3.902, 3.903, 3.892, 3.885, and 3.884 Å, respectively. The decrease of lattice parameter for PdRuPt during heat treatment may be due to the replacement of the Pt or Pd atoms on the lattice points of the f.c.c. crystal structure by the smaller Ru atoms, which will be discussed in the subsequent XPS characterization^37^.

**Figure 3:**
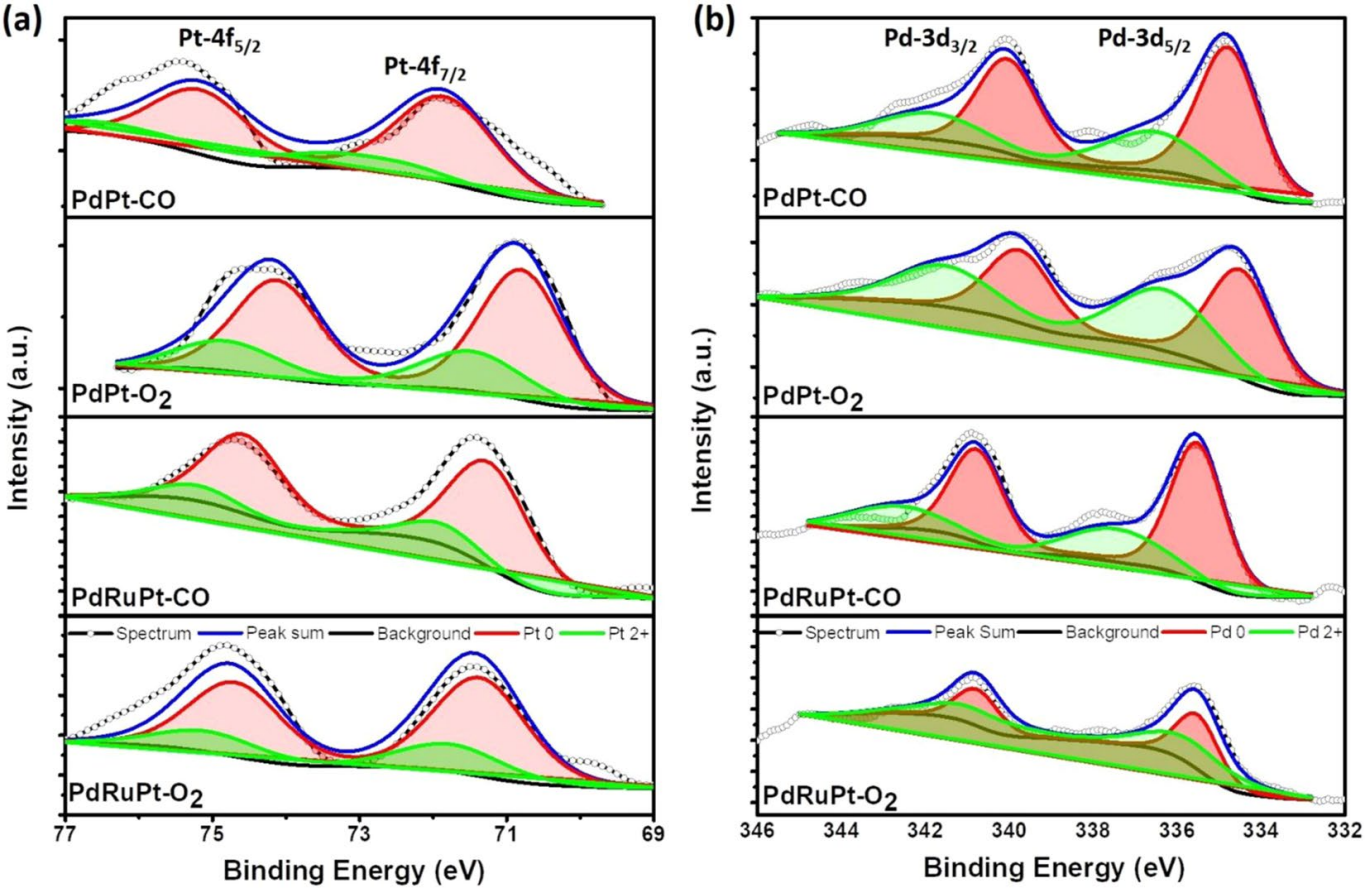
X-ray photoelectron spectroscopy of experimental NCs. (**a**) Pt-4f and (**b**) Pd-3d orbitals of PdPt-CO(O_2_) and PdRuPt-CO(O_2_) NCs.

The surface chemical composition along with binding energies of as-prepared and post-annealed NCs were investigated via X-ray photoelectron spectroscopy (XPS). Figure 3 presents the fitted XPS spectrums in the Pt-4f **(**Fig. 3a**)** and Pd-3d **(**Fig. 3b**)** regions for the post-annealed NCs. In a Pt-4f spectrum, the broad peaks centered nearly at ~71 eV and ~74 eV are corresponding to photoelectron emissions from Pt 4f_7/2_ and Pt 4f_5/2_ orbitals, respectively. Whereas for Pd-3d spectrum, the broad peaks located nearly at ~336 eV and ~341 eV are attributed to photoelectron emission from Pd 3d_5/2_ and Pd 3d_3/2_ orbitals, respectively. These emission peaks are further de-convoluted for revealing the extent of different oxidation states and corresponding results are summarized in Table 1. Accordingly, no significant change is observed in the surface composition of PdPt NCs during heat treatment. On the other hand, Pt content on the surface of PdRuPt NCs is increased. Such behavior is obvious because Pt and Pd have the same f.c.c. crystal structure, similar atomic size and lattice constant, the large amount of surface Ru observed from CO-stripping results (later section) may diffuse into Pd lattice and replace some inner Pt atoms, resulting in the Pt surface segregation. Meanwhile, it can be noticed that Pt 4f_7/2_ peak position for PdRuPt have large positively shift after CO and O_2_ treatment. The peak positions of Pt in Pt 4f_7/2_ region for as-prepared PdRuPt is located on 70.5 eV (Figure S2 and Table 1), and for PdRuPt-CO and PdRuPt-O_2_, those are located on 71.3 and 71.4 eV, respectively. This phenomenon can be due to the compressive strain introduced by increasing surface Pt with a larger lattice parameter over inner Pd and Ru with smaller lattice parameters^38^. The closer distributions of surface Pt atoms for heat-treated PdRuPt than as-prepared PdRuPt may cause the ensemble effect, and influence the electrochemical performance. As shown in Fig. 3, the Pt^2+^/Pt and Pd^2+^/Pd ratio for PdPt-O_2_ and PdRuPt-O_2_ are higher than those of PdPt-CO and PdRuPt-CO, suggesting the surface oxidation of catalysts during O_2_ treatment.

**Table 1:** XPS determined composition ratios and binding energy of experimental NCs.

Samples	Elemental Chemical States				Surface Composition (%)		Binding Energies (eV)		ECSA_CO_ (m^2^/g_Pt+Pd_)
	Pt 0	Pt 2+	Pd 0	Pd 2+	Pt	Pd	Pt 0	Pd 0	
PdPt	76	24	70	30	9	91	71.0	335.9	28.3
PdPt-CO	77	23	70	30	8	92	71.5	335.6	26.5
PdPt-O_2_	73	27	55	45	8	92	71.1	335.8	19.1
PdRuPt	80	20	71	29	10	90	70.5	335.5	54.4
PdRuPt-CO	82	18	71	29	14	86	71.3	335.5	35.3
PdRuPt-O_2_	76	24	63	37	15	85	71.4	335.7	38.2

*ECSA is determined via CO-stripping analysis.

**Figure 4:**
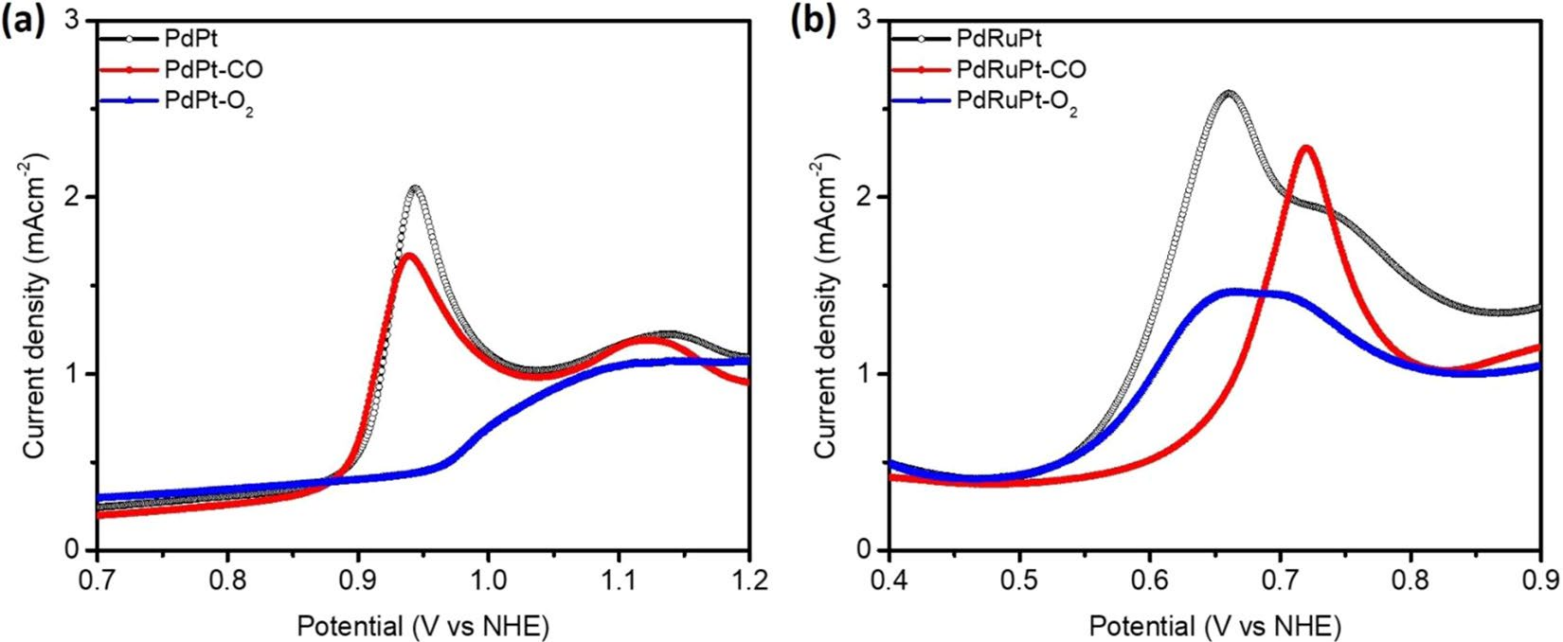
The CO-stripping voltammogram of as prepared and post-annealed NCs. (**a**) PdPt NCs and (**b**) PdRuPt NCs.

CO stripping analysis (Fig. 4) has been employed to demonstrate the capability of adsorbed CO oxidation with respect to the surface composition of experimental NCs. As depicted in Fig. 4, the CO oxidation intensity for all catalysts after heat treatment decreases owing to the increase of the particle size^39^. For PdPt-O_2_ and PdRuPt-O_2_ NCs, more severe decreasing of CO oxidation intensity can be attributed to the oxidation of Pt and Pd, as shown in XPS results (Fig. 3). The onset potentials for PdPt, PdPt-CO, PdPt-O_2_, PdRuPt, PdRuPt-CO and PdRuPt-O_2_ is located at 875, 875, 950, 510, 560, and 515 mV, respectively. In a CO-stripping curve, the positions of adsorbed CO (i.e. CO^ads^) oxidation peaks reflects the required potential to drive the maximum CO^ads^ oxidation kinetics (i.e. onset potential). For various PdPt catalysts, PdPt-CO has almost the same onset potential and peak position when compared to PdPt, indicating no obvious changes cross-referencing surface composition during CO heat treatment. Besides, the positive shift in onset potential of PdPt-O_2_ may be due to the oxidation of Pt and Pd (Table 1) which increases the onset of CO oxidation peak by 0.2 V (i.e., CO binding energy) as compared to that of PdPt (Table S1). The CO annealing slightly reduced the onset by 0.013 V which can be attributed to the segregation of Pt intermix to the Pd in NC surface. On the other hand, for various PdRuPt catalysts, positive shifts are noted for both CO and O_2_ treated samples, because Pt atoms tend to segregate to NP surface during the heat treatment. In this event, oxidation is suppressed by the CO chemisorption followed by its oxidation to CO_2_. Such a reaction results in high contents of metallic Pt and Pd therefore the CO binding energy (onset and peak potential) of PdRuPt-CO are increased. RuO_2_ and Ru are generally regarded as a beneficial species to regenerate the CO-poisoned Pt^40,41^, and higher Pt/Pd ratio of PdRuPt-O_2_ than PdPt-O_2_ may lead to the more Pt active sites of the former, resulting in the more positive shift of onset potential for the former when compared to as-prepared PdRuPt.

**Figure 5:**
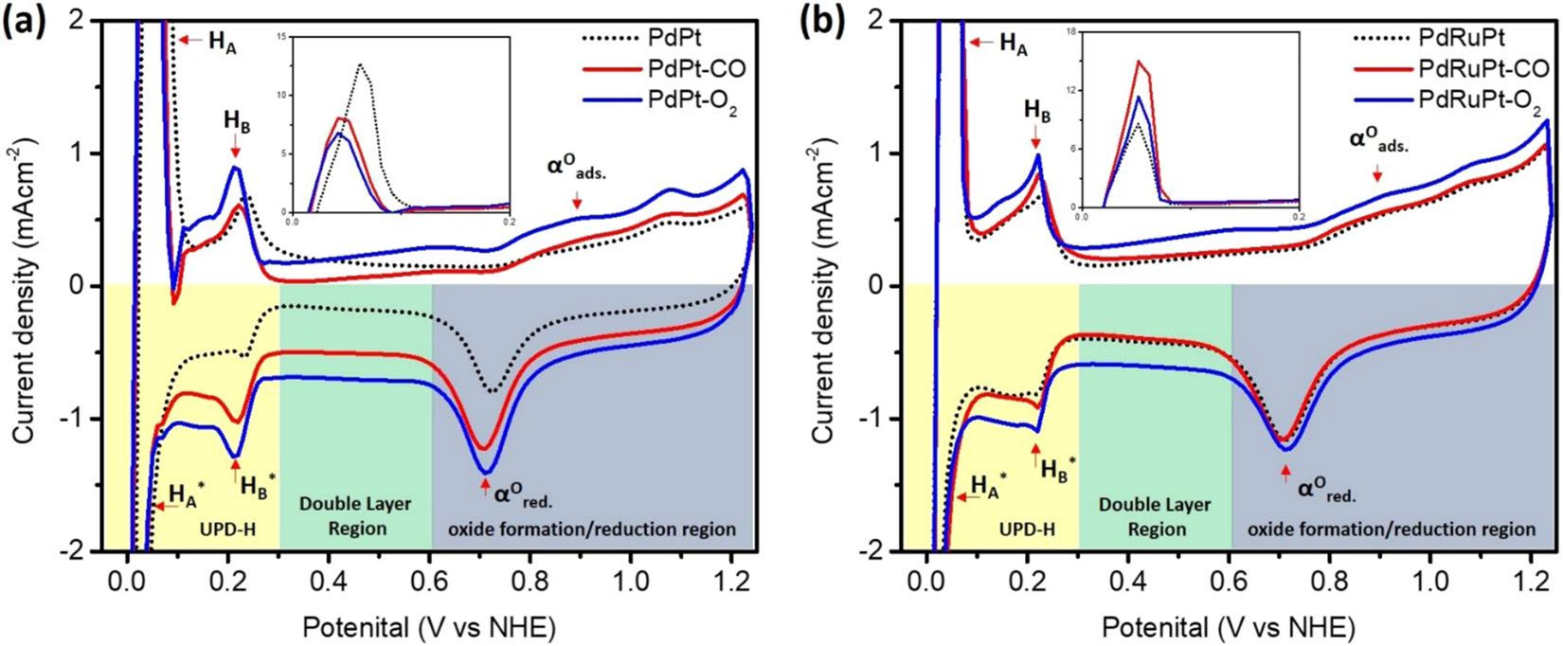
(**a**) The CV curves of as prepared and post-annealed NCs. (**a**) PdPt NCs and (**b**) PdRuPt NCs.

The CV curves (Fig. 5) were measured in an N_2_ saturated 0.5 M H_2_SO_4_ electrolyte solution at a sweeping rate of 20 mV s^−1^ under room temperature. In a CV curve, three distinctive potential regions are found, including under-potential deposition of hydrogen (UPD-H) region between 0 < E < 0.30 V, the double-layer region in between (0.30 to 0.60 V), and the chemisorption/reduction of oxygen species over 0.60 V vs. NHE. The severe variations in CV profiles of pristine and post-annealed experimental NCs are attributed to the changes in surface composition and can be observed for UPD-H (0 < E < 0.30 V vs NHE) and surface oxide regions (> 0.60 V vs NHE). As shown in the inset of Fig. 5a, the hydrogen reductive absorption and oxidative desorption peaks (H_A_ and H_A_^*^) for PdPt-O_2_ NC are significantly suppressed as compared to that of that of PdPt and PdPt-CO NCs, indicating the oxidation of PdPt NC in O_2_ atmosphere (consistent with former XPS findings), resulting in the large decrease of hydrogen adsorption sites^42^. Meanwhile, in backward scan, the oxide reduction peaks (α°_red_) for PdPt-CO and PdPt-O_2_ NCs tend to shift towards lower potentials (i.e. negative shift) as compared to that of pristine PdPt NC, reflecting higher energy barrier for reduction of oxygen species from PdPt-CO and PdPt-O_2_ NCs surface. Such a behavior is obvious due to increased index of Pd-atoms on the surface of PdPt-CO and PdPt-O_2_ NCs (Table 1). It is worth to notice that Pd has strong affinity with high selectivity pathways for oxygen adsorption. For PdRuPt NCs, the increase of hydrogen reductive absorption and oxidative desorption peak current density after heat treatment could be attributed to the decrease of surface Ru, which shows different trends when compared to CO-stripping results. According to literature, Ru would form Ru-OH_ad_ during the catalytic reaction, which will help CO oxidation. Thus, the change of surface Ru content may not influence the current density of CO oxidation significantly^43^. The surface Ru may suppress hydrogen absorption in low potential. Therefore, the increase of hydrogen region peak current could be attributed to the decrease of surface Ru, suggesting the increase of hydrogen absorption sites^44^.

All the CV curves were recorded in N_2_ saturated 0.5 M H_2_SO_4_ electrolyte solution.

**Figure 6:**
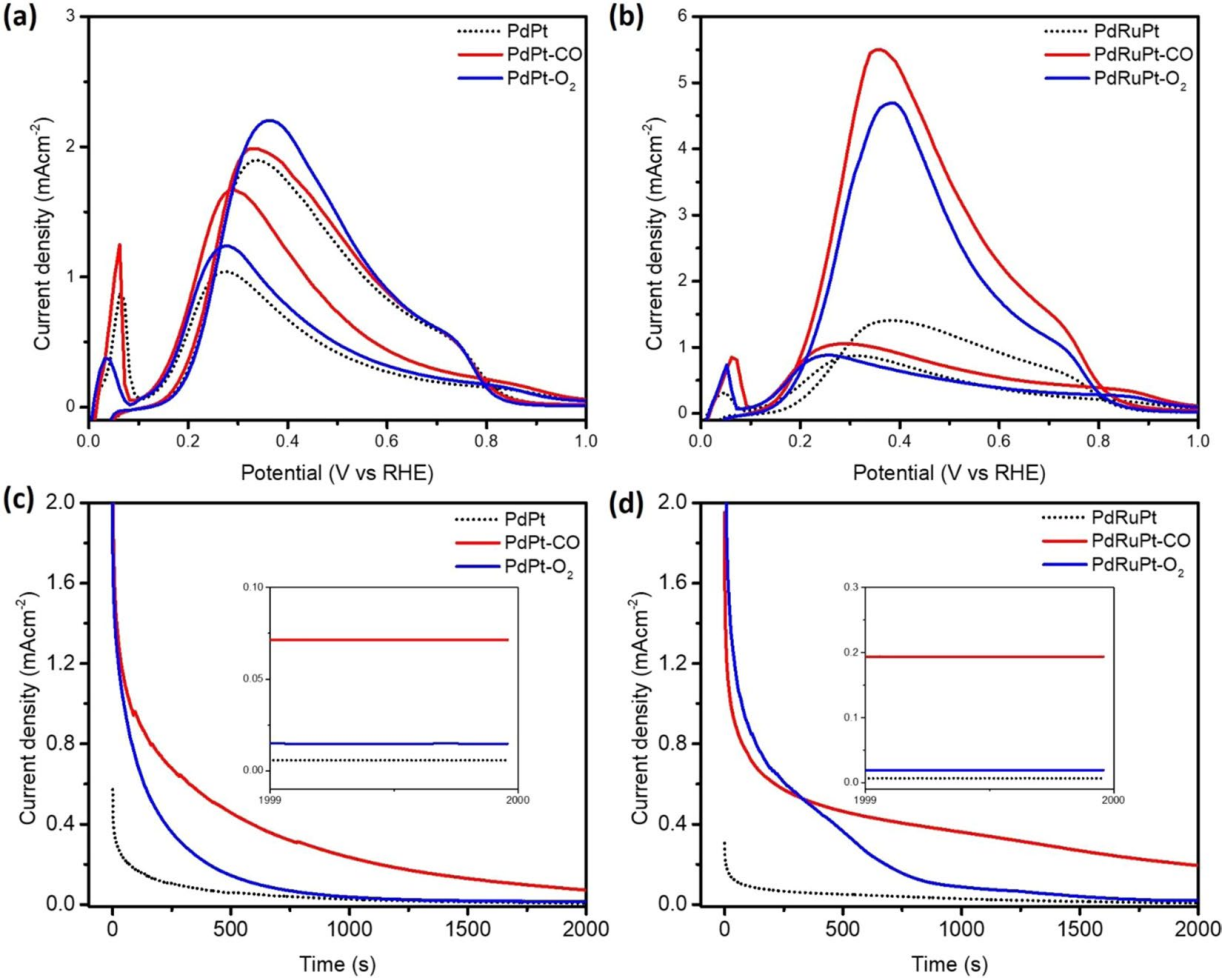
The LSV patterns of (**a**) PdPt, PdPt-CO, PdPt-O_2_ and (**b**) PdRuPt, PdRuPt-CO, PdRuPt-O_2_ NCs. (**c**) CA patterns of PdPt, PdPt-CO, PdPt-O_2_ and (**d**) PdRuPt, PdRuPt-CO and PdRuPt-O_2_ NCs. All data were measured in 0.5 M H_2_SO_4_ and 0.5 M HCOOH saturated with N_2_.

Figure 6a,b show the LSV results of pristine and post-annealed PdPt and PdRuPt NCs, respectively, measured in 0.5 M H_2_SO_4_ and 0.5 M HCOOH solution. For both pristine and post annealed experimental NCs, the onset potential measured from CO-stripping, the maximum current density in the forward and backward scan for FAOR, and the mass activity (MA) obtained when FAOR current density was normalized to the Pt + Pd loading, are summarized in Table 2. Accordingly, the MA of PdPt, PdPt-CO, PdPt-O_2_, PdRuPt, PdRuPt-CO, and PdRuPt-O_2_ NCs in the anodic/cathodic scan is 1.0/1.9, 1.7/2.0, 1.3/2.2, 0.9/1.4 1.1/5.5, and 0.9/4.7 A/mg, respectively. The significant change in electrochemical properties of PdRuPt NCs (Fig. 6b) during both CO and O_2_ treatments is attributed to change in surface compositions. The Pt-atoms tends to strongly adsorb CO_ads_ at relatively low potentials and quickly blocks the surface sites, but for Pd, the poisoning by CO_ads_ at low potentials is much slower. The Pt surface segregation in PdPt bimetallic catalyst may decrease anodic current density^45^. Thus, the much lower I_f_/I_b_ ratio for PdRuPt-CO(O_2_) as compared to that of as-prepared PdRuPt (0.6) may be due to an increase of surface Pt/Pd ratio which leads to ensemble effect, and meanwhile the decrease of surface Ru can highly enhance the total oxidation activity. Meanwhile, for PdPt NCs, the catalytic current did not change significantly after heat treatment compared to PdRuPt NCs, is attributed to the smaller change of surface composition as that of PdRuPt NCs, which is consistently proved by former XPS and CO-stripping characterizations.

**Table 2:** Electrochemical results of pristine and post-annealed experimental NCs.

Samples	E^a^_onset_	I_f_ (mAcm^−2^)	I_b_ (mAcm^−2^)	MA_f_ (Pt&Pd) (A/mg)	MA_b_ (Pt&Pd) (A/mg)	MA_f_ 05–2000 s (mA/mg)
PdPt	0.875	9.9	18.0	1.0	1.9	54
PdPt-CO	0.875	15.6	18.3	1.7	2.0	72
PdPt-O_2_	0.950	11.4	20.4	1.3	2.2	15
PdRuPt	0.510	7	11.2	0.9	1.4	62
PdRuPt-CO	0.560	8.2	42.7	1.1	5.5	213
PdRuPt-O_2_	0.515	6.8	36.4	0.9	4.7	22

^a^ Calculated from CO stripping analysis.

In order to characterize the durability of different catalysts, CA test was employed in N_2_-saturated 0.5 M H_2_SO_4_ and 0.5 M HCOOH solution (Fig. 6c,d). After CA test up to 2000s, PdRuPt-CO exhibits the highest activity, because moderate surface Ru content can remove adsorbed CO_ads_ and increase CO tolerance of catalysts, and enhance FAO durability^46^. For PdPt-O_2_ and PdRuPt-O_2_, both samples show lower activity after CA than CO treated samples, attributed to severe oxidation of catalyst deteriorating the durability.

Furthermore, the evolution of NC’s surfaces during CA test of experimental NCs can be elucidated by the CV results. As shown in Figure S3. During the stability test of PdPt-CO and PdPt-O_2_, the cathodic peak potential does not shift significantly, suggesting any obvious change in surface compositions, and the decrease of peak intensity in the hydrogen region may be due to agglomeration of NPs. For PdRuPt-CO and PdRuPt-O_2_, the current density in the hydrogen region almost keeps the same after CA, indicating that Ru can prevent the decrease of hydrogen adsorption sites during the stability test.

**Scheme 1:**
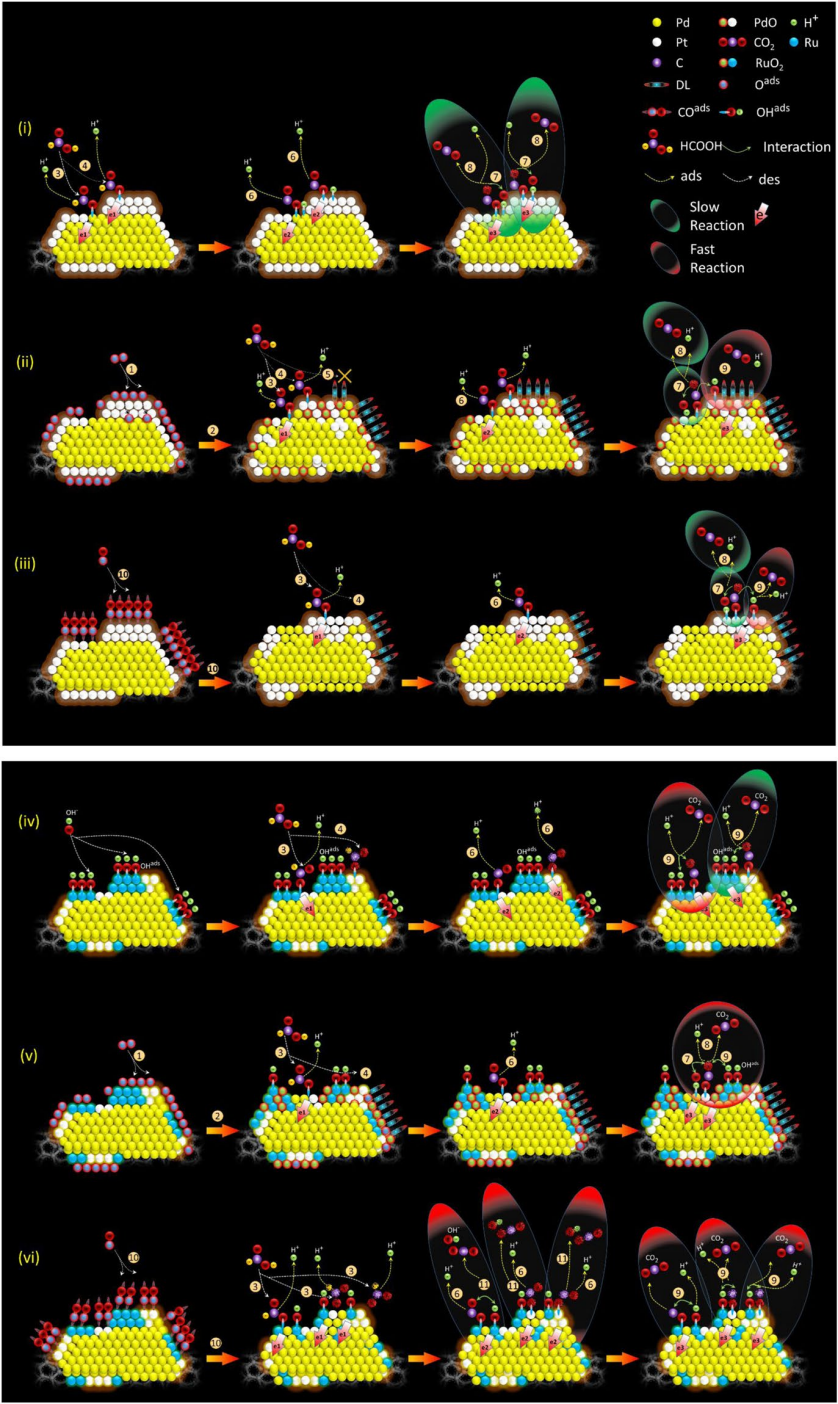
Schematic representation for the possible reaction pathways for formic acid oxidation on the samples of (i) PdPt, (ii) PdPt-O_2_, (iii) PdPt-CO, (iv) PdRuPt, (v) PdRuPt-O_2_, and (vi) PdRuPt-CO.

By cross-referencing results of physical inspections and electrochemical analysis, we can figure out the annealing induced atomic restructure and their corresponding impacts on the formic acid oxidation (FAO) performance of the experimental NCs. Accordingly, the higher double-layer current density in CV sweeping curves (Fig. 5a) of PdPt-CO and PdPt-O_2_ NCs compared to as prepared PdPt NC is observed. Such an enhancement in double-layer capacitance for PdPt-O_2_ NC can be rationalized due to the severe oxidation of Pt and Pd-atoms (consistently proved by the positive shift in onset potential of PdPt-O_2_ in CO-stripping curve) in oxygen atmosphere (Table 1). Meanwhile, in CO atmosphere (i.e. for PdPt-CO NC), although the double-layer capacitance is significantly increased compares to as-prepared PdPt NC, however, attenuated when compared to PdPt-O_2_ NC. Such a behavior is attributed to the segregation of Pt intermix to the Pd on NC surface together with increased content of carbon support^47^. On the other hand, there is no significant change observed in the double-layer capacitance after CO ambient treatment (i.e. for PdRuPt-CO) when compared to as prepared PdRuPt NC, suggesting the oxidation is suppressed by the CO chemisorption followed by its oxidation to CO_2._ Whereas, PdRuPt-O2 NC exhibits similar behavior as that of PdPt-O_2_ NC. Those structure evolutions end up with different reaction pathways on the FAO reaction in the experimental NCs and the corresponding results are summarized in Scheme 1 where details are breakdown in 11 equations:

1. Pd* + O_2(g)_ → Pd*-O_2_^ads^ + Pd* → 2 Pd*-O^ads^
2. Pd*-O^ads^ → Δ → PdO
3. Pt* + HCOOH → Pt*-OOCH^ads^ + H^+^ + e1… (fast)
4. Pd* + HCOOH → Pd*-OOCH^ads^ + H^+^ + e1… (**slow**)
5. PdO + HCOOH → X
6. Pt*-OOCH^ads^ → Pt*-OOC^ads^ + H^+^ + e2
7. Pt*-OOC^ads^ + Pt*-H^+^ → Pt*-CO^ads^ + Pt*-OH^ads^
8. Pt*-OC^ads^ + Pt*-OH^ads^ → Pt* + Pt* + CO_2_ + H^+^ + e3 (**very slow**)
9. Pt*-OC^ads^ + (Ru*-OH^ads^ /Pd*-OH^ads^) → Pt* + (Ru* / Pd*)+ CO_2_ + H^+^ + e3 (**fast / slow**)
10. Pt@Pd* + CO_(g)_ → Pt@Pd*-CO^ads^ → Δ → Pd@Pt-CO^ads^ (Pt segregation)
11. Pt*-OOC^ads^ + Ru*-OH^ads^ → Pt*-CO^ads^ + RuO*-OH^ads^ → Pt* + RuO* + CO_2_ + OH- (**slow**)
12. Pt@RuO* + H^+^ → Pt@RuO*-H^ads+^ → Pt@Ru*-OH^ads+^ → Pt@Ru*^+^ + OH^-^
13. Pt@Ru*^+^ + H^+^ → Pt@Ru^2+^*-H^ads^ + OH^-^ → Pt* + Ru^3+^ + H_2_O

these reaction pathways complimentary explain the FAO performances of the experimental samples. In the O_2_ ambient, the 1^st^ and 2^nd^ pathways segregate and oxidize Pd to PdO in PdPt-O_2_ surface (model (ii)). With the formation of PdO, the adsorption of OH ligands so as to the coverage of double-layer (DL) region on the surface is increased (the 2^nd^ step). Formation of Pd oxide is complimentary proved by the strong CO adsorption bond with the increments of 0.098 volt for onset potential and 0.2 volt for peak position on the desorption peak in the CO stripping curve. Such a scenario reduces the number of active sites (Pt atom). In this event, even with the increase of OH^ads^, the FAO performance of PdPt-O_2_ can only slightly be improved as compared to that of PdPt. This characteristic can be explained by the reduction of the number of reaction sites in eqn 3 accompanied with the increase of reaction sites in eqn 4 and its subsequent eqn 8 in PdPt-O_2_ as shown by the 2^nd^ to 4^th^ steps in the model (ii)). Meanwhile, these surface oxides are unstable in the acid electrolyte, therefore, the FAO current density of PdPt-O_2_ is dramatically decreased in the CA test. On the other hand, CO adsorption segregates and oxidize Pt atoms to NP surface. This scenario increases the number of active sites and reinforces the crystal structure (the 1^st^ step (10^th^ pathway) of the model (iii)), therefore, improves both the current density and durability of PdPt-CO in FAO performance (the 3^rd^ to 4^th^ step in the model (iii)). Tri-metallic nanocatalysts shown different responses on physical characteristics so as to the electrochemical properties to those of PdPt upon annealing in O_2_ and CO ambient. For PdRuPt, oxidation treatment segregates Ru and Pd atoms to the PdRuPt-O_2_ surface (the 1^st^ and 2^nd^ pathways in the 2^nd^ step of model (v)). In this event, the prevailing OH chemisorption (OH^ads^, see step 2 and 3 in the model (v)) in Ru and Pd oxides suppresses the CO binding energy (decrease of onset by 0.007 volt) and thus increases the activity of Pt sites to improve the current density of PdRuPt-O_2_ as compared to that of PdRuPt. In the CA test, the same scenario to that of PdPt-O_2_ hold therefore a dramatical suppression of the FAO performance is expectable in PdRuPt-O_2_ (eqn 12 and eqn 13 not shown in Scheme 1). Compared to that of PdRuPt, CO adsorption triggers the Pt segregation and reduction to the surface and are revealed by a positive shift of CO stripping curve in PdRuPt-CO. By annealing in CO (model (vi)), Pt segregation (10^th^ pathway in the 1^st^ step) and Ru hydration collaborate in the NP surface. It not only increases the intermix between Pt and neighboring atoms (Ru and Pd) but reduces the local disorder to reinforce the durability and activity (via pathway 9^th^ in model (vi)) of PdRuPt-CO in CA.

## Conclusion

We develop a ternary PdRuPt catalyst with ultra-low Pt content (~2 at%) and a FAOR performance higher than pure Pt catalysts. Meanwhile, the FAOR performance is further enhanced by more than 30% via a unique CO annealing treatment demonstrated in this study. The samples of carbon-supported Pd-based binary (PdPt) and ternary (PdRuPt) NCs were prepared via the simple polyol reduction method followed by the heat treatment in CO and O_2_ atmosphere at 473 K. By cross-referencing results of the physical structure and electrochemical inspections, we demonstrate that due to severe oxidation, the MA of O_2_ treated PdPt and PdRuPt NCs is promoted but the stability is deteriorated by 10–25% as compared to pristine samples. Whereas, by adopting the unique annealing in CO ambient, both the PdPt and PdRuPt NCs show an improved current density by 30% as-prepared ones. Such an enhancement is attributed to the increase of surface Pt contents and intermix between neighboring atoms, especially for PdRuPt NC. With the co-existence of Pt and Ru, Pd atoms on the surface, the collaboration between reaction sites facilitates the oxidation of formic acid accompanied by the CO oxidation reactions. The collaboration of the two pathways regenerate the active sites (Pt) and thus improves reaction kinetics and reduces the chemical loading of NCs in FAOR. Aforementioned scenarios simultaneously improve the activity and durability of PdRuPt in a CA test.

## Materials and Methods

### Preparation of experimental NCs

The Pd-based binary and ternary NCs were prepared via the modified polyol reduction method^48^. In the first step, For the preparation of Pd nanoparticles (NPs) with metal loading about 10 wt. %, 20 g of ethylene glycol (EG, Showa chemical Co.Ltd.) solution containing 10 wt. % of polyvinylpyrrolidone (PVP, MW = 10000, Aldrich) and 443 mg of Palladium (II) chloride (PdCl_2_; Pd, 99%, Sigma-Aldrich Co.) was mixed in a flask. The mixture was kept at 160 °C for 2 h until Pd precursor was completely reduced. As obtained solution was cooled at room temperature and dark colloid was observed, indicating the formation of Pd NPs. In the second step, 0.25 g of as-prepared Pd NPs solution was added in a flask along with 12.5 g of 10 wt. % PVP/EG solution, 9.95 g of EG and 88.66 mg of PdCl_2_. The resulting solution was mixed and kept at 160 °C for 2 h. In the next step, the obtained solution was cooled at room temperature and washed with acetone several times to remove the solvents. Subsequently, as-prepared Pd NPs were mixed with carbon black and added to an aqueous solution (10 mL) containing 1 mL of 0.5 M H_2_SO_4_ and 1 mL of 1.0 M acetic acid. The solution was stirred vigorously at room temperature for 24 h. The resulting powder was washed with an adequate amount of acetone several times. Finally, the obtained precipitate was washed with sodium borohydride (NaBH_4_)/tert-Butylamine (TBA) solution to remove PVP. As obtained NC is named as “Pd NPs” in the following of this article.

After preparation of Pd NPs, binary (PdPt) and ternary (PdRuPt) NCs have been prepared. A similar method was adopted for the preparation of PdPt and PdRuPt NCs. For the synthesis of PdPt and PdRuPt NCs, In the first step Pt NPs were synthesized by using a similar method, which is used for the preparation of Pd NPs in the previous section. The Pt NPs were prepared by heating a mixture of 1295 mg H_2_PtCl_6_·6H_2_O (99%, Sigma-Aldrich Co.) and polyvinylpyrrolidone (PVP; MW = 10000; 10 wt.%) dissolved in 20 g of ethylene glycol (EG, Showa chemical Co.Ltd.) solvent at 160 °C for 2 h^47^. Further PdRuPt NCs were synthesized by adding 0.25 g of as-prepared Pt NPs solution in a flask along with 12.5 g of 10 wt. % PVP/EG solution, 9.95 g of EG, and 44.33 mg of PdCl_2_ (in this step amount of Pt-atoms is too less as compared of that of Pd and thus Pt-atoms tend to deposited on the surface of Pd NPs). The resulting solution was mixed and heated at 160 °C for 2 h. In a subsequent step, the obtained solution was cooled at room temperature and 51.8 mg of RuCl_3_ was added (For the preparation of binary (i.e. PdPt) NCs we did not add RuCl_3_ precursor in this step). Afterwards, the solution was heated again at 160 °C for 2 h until all metal precursors were completely reduced. The resulting colloidal solution was cooled at room temperature and washed with acetone several times to remove the solvents. Finally, the as-prepared NPs and carbon black were added to the aqueous solution (10 mL) containing 1 mL of 0.5 M H_2_SO_4_ and 1 mL of 1 M acetic acid. The solution was stirred vigorously at room temperature for 24 h. The resulting powders were precipitated and washed with acetone several times. Then the catalysts were washed with NaBH_4_/ TBA solution to remove PVP and named as PdRuPt samples. As-prepared NCs were further subjected to heat treatment at 473 K in CO or O_2_ atmosphere with a flow rate of 100 mL min^−1^. The CO-treated and O_2_-treated PdPt were designated as PdPt-CO and PdPt-O_2_, respectively, and the CO-treated and O_2_-treated PdRuPt were designated as PdRuPt-CO and PdRuPt-O_2_, respectively. Detailed flowcharts for synthesis procedure of Pd, PdPt and PdRuPt NCs have been added in supplementary information (Figure S4 and Figure S5).

### Physical characterization

The average particle size and surface morphologies of pristine NCs and after Chronoamperometric (CA) stability tests were inspected by using High-resolution transmission electron microscope (HRTEM) (JEOL JEM 2100 F). The electron gun is a LaB6 model and is operated at an accelerating voltage of 200 kV. Details of sample preparation and analysis were reported in a previous study^49^. The crystal structure of the NCs were determined by using X-ray diffraction (XRD) analysis. The diffraction patterns of experimental NCs were measured in a 2θ range from 20° to 80° at a scan rate of 0.0124° s^−1^ by using a commercial diffractometer (Rigaku, Cu Kα (λ = 1.54 Å), operating at 40 kV and 40 mA). The X-ray photoelectron spectroscopy (XPS) spectra were measured by commercial available analyzer (Thermo VG Scientific Sigma Probe, operated at a voltage of 20 kV and a current of 30 mA) with a monochromatic X-ray source (Al Kα). The surface compositions of the catalysts were determined by fitting the integral of emission lines. Before peak fitting, Shirley type background was adopted for eliminating the noise from the inelastic electrons. After subtraction, a combination of Lorentzian and Gaussian lines was applied for peak analysis. The binding energies were calibrated by referencing to the standard energy of C 1 s peak (284.6 eV). The compositions of the experimental NCs were estimated by using ICP-AES (Jarrell-Ash, ICAP 9000) analysis. Accordingly, the atomic ratios are 86/10/4 for Pd/Ru/Pt, 100/0/0 for Pd, and 98/0/2 for PdPt.

### Preparation of the electrode and electrochemical measurements

Electrochemical analysis was conducted at room temperature (25 ± 1 **°**C) by using a commercial potentiostat (CH Instruments Model 700 A, CHI 700 A). The experimental samples were placed in the working electrode of a regular three-electrodes electrochemical cell^48^. Catalyst ink was prepared by dispersing 5.0 mg of catalyst powder in 1.0 ml of isopropanol (IPA) and 50 μl of Nafion (5 wt.%, DuPont) with a ultra-sonication for 30 minutes. In the electrochemical test, 10.0 μl of catalyst ink was drop-casted and air-dried on a working electrode of glassy carbon rotating disk electrode (RDE) (0.196 cm^2^ area). A saturated calomel electrode (SCE) and a Pt wire were respectively used as a reference and counter electrodes. Potentials of all sweeping curves were calibrated to the normal hydrogen electrode (NHE).

For evaluating the FAOR performances, the aqueous solution of 0.5 M H_2_SO_4_ + 0.5 M HCOOH mixture was used as the electrolyte and was saturated by highly-purified N_2_ at room temperature. Prior to the test, the electrodes were cycled several time between 0 and 1.4 V, to produce clean surfaces at a scan rate of 50 mV s^−1^. Formic acid oxidation current was measured by linear sweep voltammetry (LSV) at a potential sweeping rate of 20 mV s^−1^ between 0 and 1.05 V. The mass activity (MA) was calculated by using the following equations:

3 $$MA(Pt+Pd)=\frac{1}{[{\rm{Pt}}]+[{\rm{Pd}}]}$$

Where I represented the current density and [Pt] + [Pd] represented the Pt and Pd loading (mg cm^−2^) on the electrode, respectively. All current densities are normalized by the electrode area and metal loading on the electrode. The cyclic voltammetry (CV) plots were measured at a scan rate of 20 mV s^−1^ in a N_2_-saturated 0.5 M H_2_SO_4_ aqueous solution between 0.0 and 1.2 V vs. NHE^48^. The chronoamperometric (CA) test, was conducted at the potential of 0.5 V for 2000 s. For the CO stripping test, the working electrodes with NCs were immersed in a CO purged electrolyte (0.5 M H_2_SO_4_) at 0.34 V for 60 min. Afterwards, the CO stripping voltammetry was measured between −0.1 and 1.20 V in N_2_ saturated 0.5 M H_2_SO_4_ solution and the potential scan rate is 50 mVs^−1^.

## Supplementary information

Supplementary Information.
