# Supplementary material for: CO-Reductive and O2-Oxidative Annealing Assisted Surface Restructure and Corresponding Formic Acid Oxidation Performance of PdPt and PdRuPt Nanocatalysts
Source: Sci Rep. 2020 May 21;10:8457. doi: 10.1038/s41598-020-65393-3 (PMC7242419; doi:10.1038/s41598-020-65393-3)
Supplement: Supplementary file 1 — Supplementary Information. [file 41598_2020_65393_MOESM1_ESM.docx]

**CO-Reductive and O_2_-Oxidative Annealing Assisted Surface Restructure and Corresponding Formic Acid Oxidation Performance of PdPt and PdRuPt Nanocatalysts**

Authors: Dinesh Bhalothia,^a^ Tzu-Hsi Huang,^b^ Pai-Hung Chou,^b^ Po-Chun Chen,^c^ Kuan-Wen Wang,^b*^ and Tsan-Yao Chen^a,d*^

Affiliations:

^a.^ Department of Engineering and System Science, National Tsing Hua University, Hsinchu 30013, Taiwan.

^b.^ Institute of Materials Science and Engineering, National Central University, Taoyuan City 32001, Taiwan.

^c.^ Department of Materials and Mineral Resources Engineering, National Taipei University of Technology, Taipei 10608, Taiwan

^d.^ Hierarchical Green-Energy Materials (Hi-GEM) Research Centre, National Cheng Kung University, Tainan 70101, Taiwan

Corresponding Authors:

Tsan-Yao Chen

Email: [chencaeser@gmail.com](mailto:chencaeser@gmail.com)

Tel: +886-3-5715131 # 34271

FAX: +885-3-5720724

Kuan-Wen Wang

Email: kuanwen.wang@gmail.com

Tel: +886-3-4227151 # 34906

**1. HRTEM images of the as prepared (a) PdPt and (b) PdRuPt NCs.**


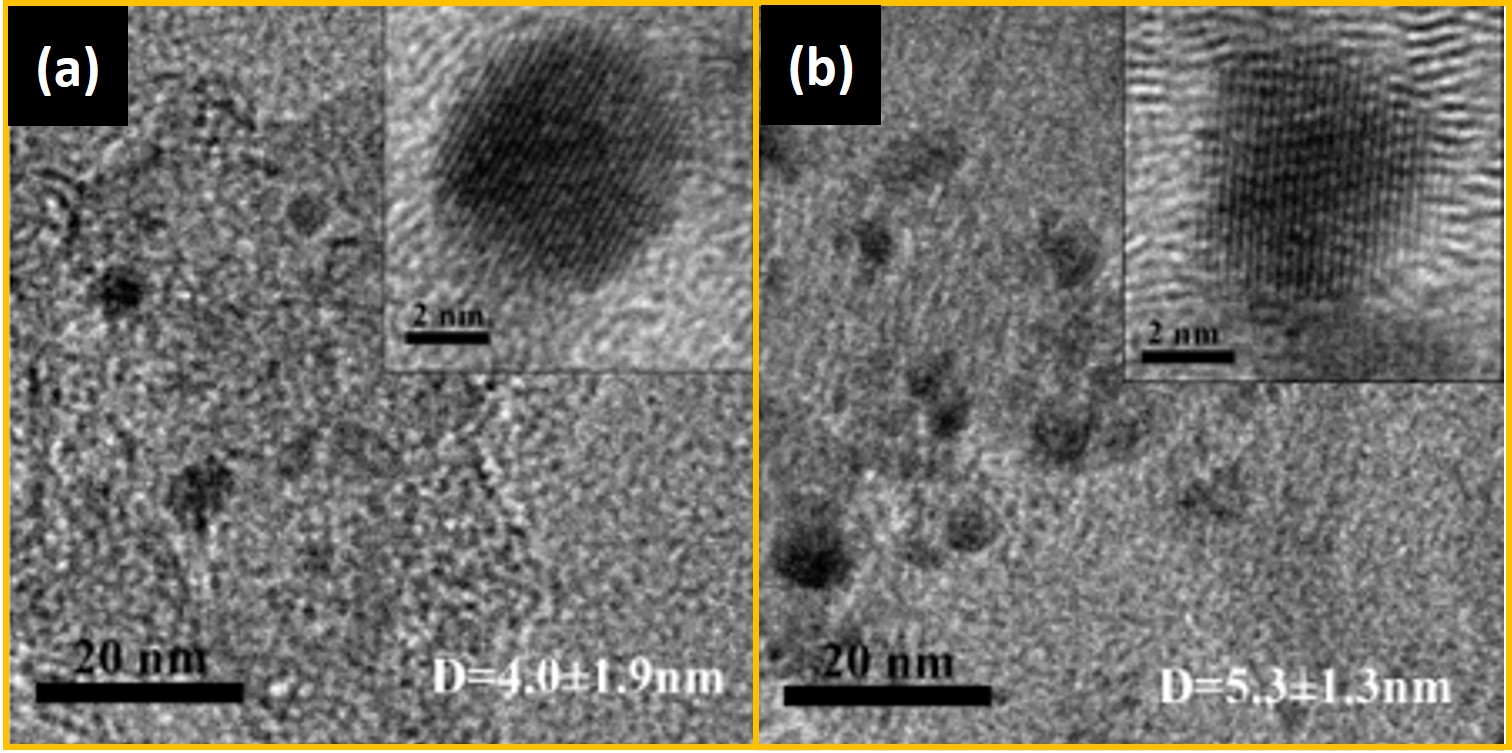


**Figure S1**. HRTEM images of the as prepared (a) PdPt and (b) PdRuPt NCs.

**2. X-ray photoelectron spectroscopy of experimental NCs. (a) Pt-4f and (b) Pd-3d orbitals of PdPt and PdRuPt NCs.**


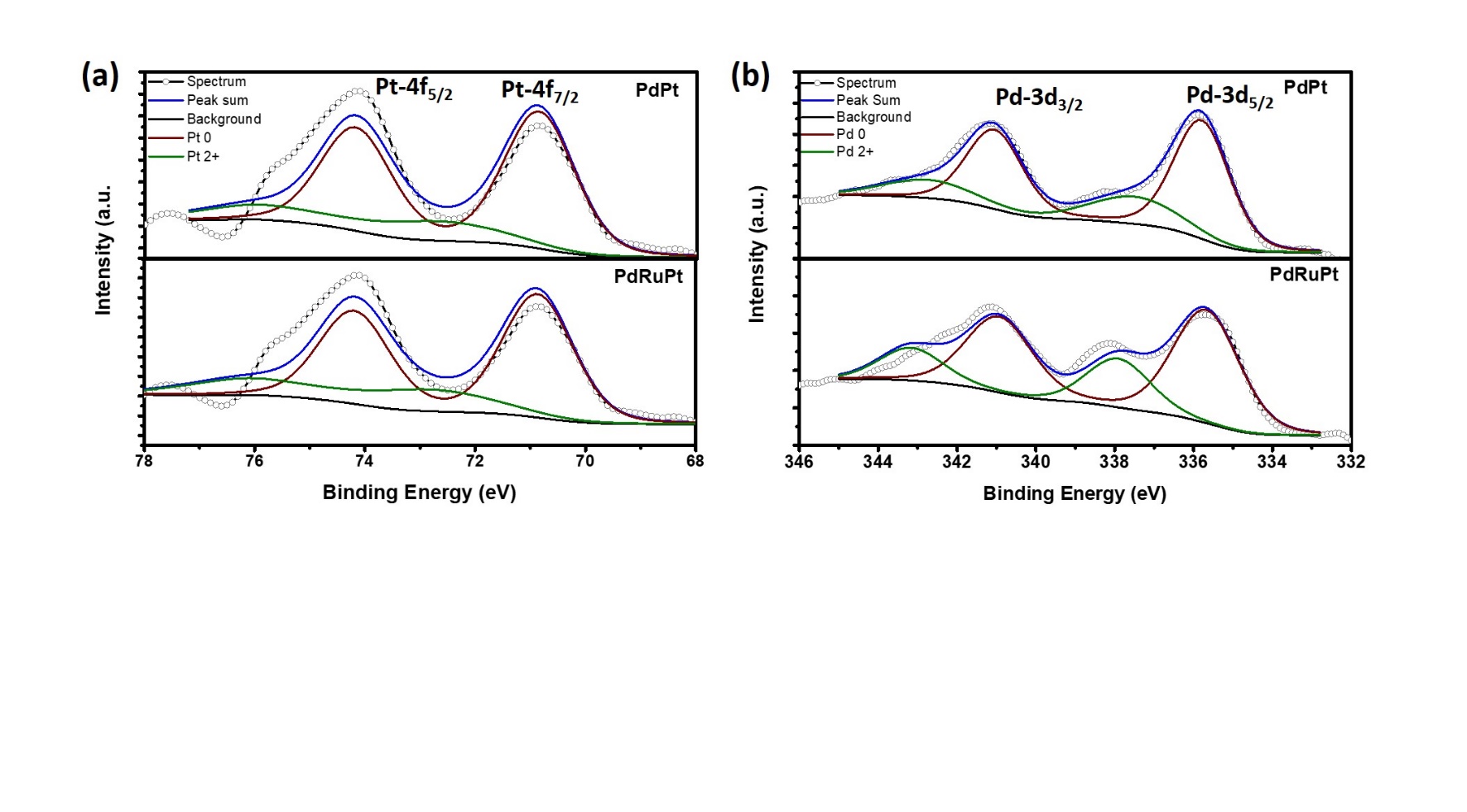


**Figure S2.** X-ray photoelectron spectroscopy of experimental NCs. (a) Pt-4f and (b) Pd-3d orbitals of PdPt and PdRuPt NCs.

**3. CV curves after CA test in formic acid oxidation reaction**.


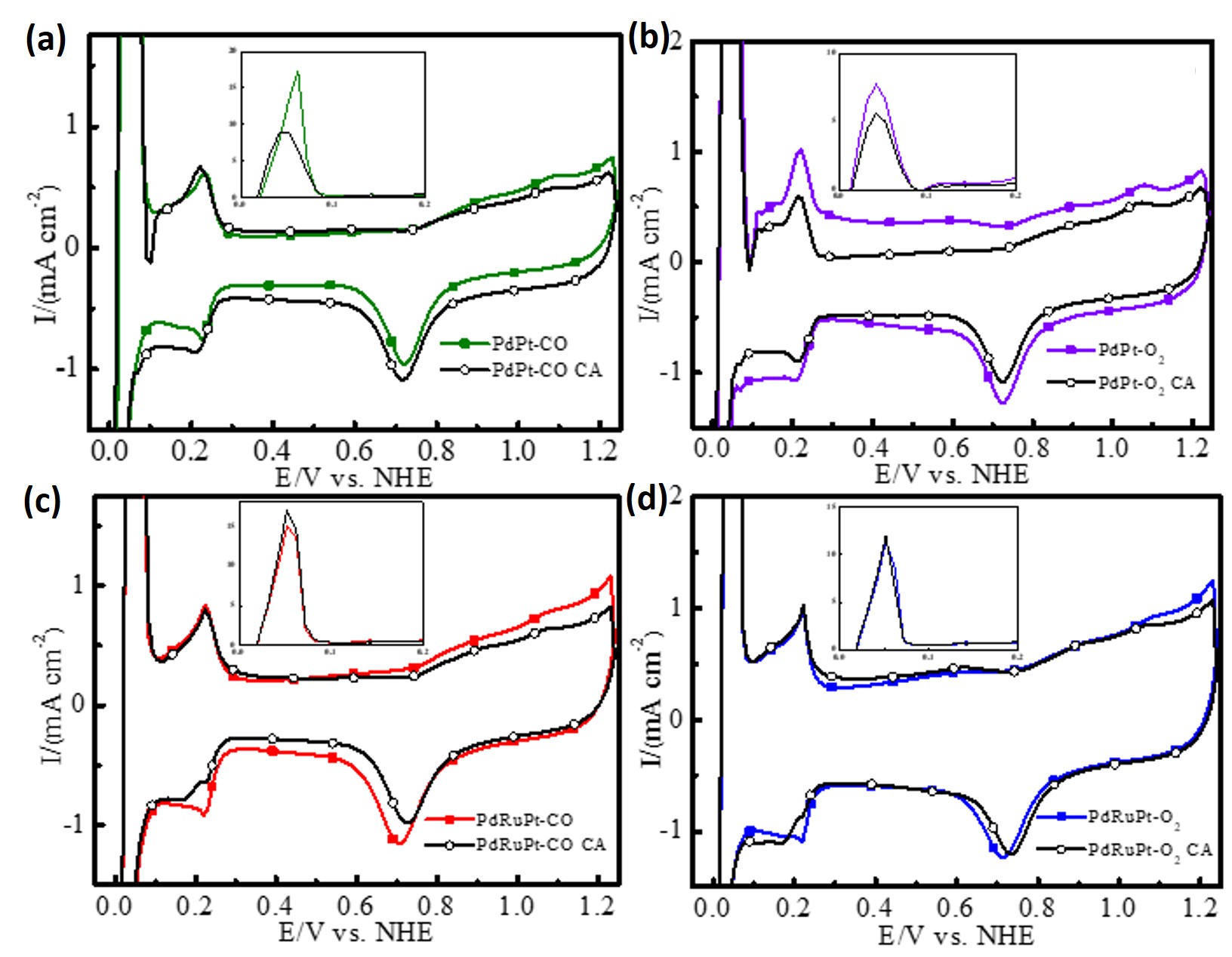


**Figure S3**. The CV sweeping curves of experimental samples after CA analysis for (a) PdPt-CO, (b) PdPt-O_2_, (c) PdRuPt-CO and (d) PdPt-O_2_ recorded in 0.5 M H_2_SO_4_ saturated with N_2._

**4. CO stripping analysis**

**Table S1**. Comparison results for the CO stripping curves for all experimental PdPt and PdRuPt nanocatalysts.

|  | Potential (V vs. NHE) | | | ΔV vs fresh (volt) | | |
| --- | --- | --- | --- | --- | --- | --- |
| sample | onset | peak | width | onset | peak | width |
| PdPt | 0.846 | 0.944 | 0.184 |  |  |  |
| PdPt-O2 | 0.944 | 1.144 | 0.213 | 0.098 | 0.2 | 0.029 |
| PdPt-CO | 0.833 | 0.939 | 0.197 | -0.013 | -0.005 | 0.013 |
| PdRuPt | 0.466 | 0.661 | 0.395 |  |  |  |
| PdRuPt-O2 | 0.459 | 0.663 | 0.359 | -0.007 | 0.002 | -0.036 |
| PdRuPt-CO | 0.538 | 0.72 | 0.281 | 0.072 | 0.059 | -0.114 |

**5. The flowcharts for the synthesis of experimental NCs.**


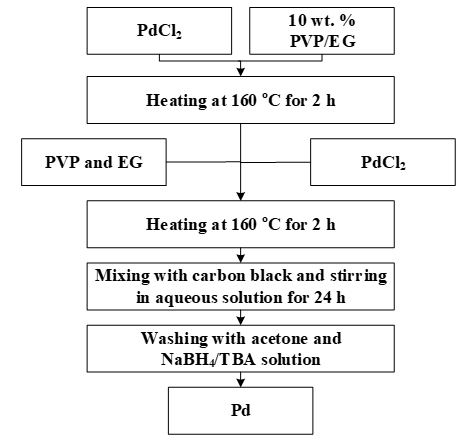


**Figure S4**. The flowchart for the synthesis of Pd NPs.


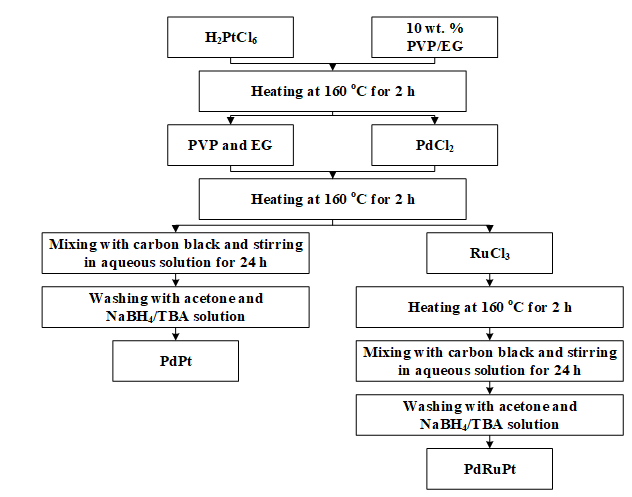


**Figure S5**. The flowchart for the synthesis of PdPt and PdRuPt NCs.
